# Supplementary material for: Analysis of PPARγ Signaling Activity in Psoriasis
Source: Int J Mol Sci. 2021 Aug 10;22(16):8603. doi: 10.3390/ijms22168603 (PMC8395241; doi:10.3390/ijms22168603)
Supplement: Supplementary file 1 [file ijms-22-08603-s001.zip › Supplemental materials_Analysis of PPARg signaling activity in psoriasis/Pathway models/Models images and html files/Interleukin-17 and interleukin-22 signaling in psoriasis/10873.html]

SPI1


# Protein SPI1

|  |  |
| --- | --- |
| URN | urn:agi-llid:6688 |
| Total Entities | 0 |
| Connectivity | 2117 |
| Name | SPI1 |
| Description | Spi-1 proto-oncogene |
| Notes | This gene encodes an ETS-domain transcription factor that activates gene expression during myeloid and B-lymphoid cell development. The nuclear protein binds to a purine-rich sequence known as the PU-box found near the promoters of target genes, and regulates their expression in coordination with other transcription factors and cofactors. The protein can also regulate alternative splicing of target genes. Multiple transcript variants encoding different isoforms have been found for this gene. [provided by RefSeq, Jul 2008] |
| Primary Cell Localization | Nucleus |
| Class | Transcription factor |
| ObjectType | Protein |

---

|  |  |
| --- | --- |
| Pathway | Thrombopoietin Receptors Signaling in Platelet Maturation |
|  | Immunoglobulin Genes Transcriptional Activation |
|  | Proplatelet Maturation |
|  | Hematopoietic Cell Lineage: B-cell (mouse) |
|  | Hematopoietic Cell Lineage: B-cell |
|  | IL17 Signaling in Psoriasis |
|  | Proteins Involved in Acute Myeloid Leukemia |
|  | RARA Signaling in Acute Myeloid Leukemia (M3) |
|  | Acute Myeloid Leukemia |
|  | Myeloid Cell Differentiation Block in Leukemia |
|  | Hodgkin Lymphoma |
|  | Hodgkin and Reed-Sternberg Cells Reprogramming |
|  | Proteins Involved in Osteopetrosis |
|  | Thrombopoietin -> SPI1 Expression Targets |
|  | Possible Interplay between SOX11, CCND1 and EZH2 in Mantle Cell Lymphoma |
|  | 2\_Interleukin-17 and interleukin-22 signaling in psoriasis |
|  | 4\_Inflammatory mediators inhibit GATA signaling in erythropoiesis |
|  | Interleukin-17 and interleukin-22 signaling in psoriasis |

---

|  |  |
| --- | --- |
| Group | Genes with Mutations Associated with Acute Myeloid Leukemia |

---

|  |  |
| --- | --- |
| MedScan ID | 6688 |
|  | 634396 |

---

|  |  |
| --- | --- |
| LocusLink ID | 6688 |
|  | 20375 |
|  | 366126 |

---

|  |  |
| --- | --- |
| Alias | Oncogene SPI I |
|  | Dis I |
|  | LOC100503306 |
|  | Tfpu.1 |
|  | SPI1 |
|  | spleen focus forming virus (SFFV) proviral integrating oncogene spi I |
|  | Tcfpu1 |
|  | hCG\_25181 |
|  | Oncogene SPI1 |
|  | RP23-20F9.1 |
|  | spleen focus forming virus (SFFV) proviral integration oncogene |
|  | Sfpi1 |
|  | 31 kDa transforming protein |
|  | Sfpi I |
|  | SFFV proviral integration 1 |
|  | SFFV proviral integration I |
|  | spi-a |
|  | OTTMUSP00000015228 |
|  | Tcfpu I |
|  | PU.1 |
|  | SPI-1 proto-oncogene |
|  | SPI I |
|  | Dis1 |
|  | spleen focus forming virus (SFFV) proviral integration oncogene spi1 |
|  | spleen focus forming virus proviral integration oncogene spi1 |
|  | SFFV proviral integrating 1 |
|  | PU.1 transcription factor |
|  | spleen focus forming virus (SFFV) proviral integrating oncogene spi1 |
|  | hematopoietic transcription factor PU.1 |
|  | spleen focus forming virus (SFFV) proviral integration oncogene spi I |
|  | PU1\_HUMAN |
|  | SFFV proviral integrating I |
|  | Transcription factor PU.1 |
|  | OF |
|  | SPI-1 |
|  | Dis-1 |
|  | Sfpi-1 |
|  | 31 kDa-transforming protein |
|  | SFFV proviral integration 1 protein |

---

|  |  |
| --- | --- |
| GO ID | 0001228 |
|  | 0003700 |
|  | 0000981 |
|  | 0001227 |
|  | 0051525 |
|  | 0003723 |
|  | 0000980 |
|  | 0000978 |
|  | 0001085 |
|  | 0008134 |
|  | 0060033 |
|  | 1902262 |
|  | 0030154 |
|  | 0071361 |
|  | 0030218 |
|  | 0030851 |
|  | 0043966 |
|  | 0044027 |
|  | 0070102 |
|  | 0030098 |
|  | 0002320 |
|  | 0030225 |
|  | 0043011 |
|  | 0045347 |
|  | 0045814 |
|  | 0090241 |
|  | 0000122 |
|  | 0045892 |
|  | 1902895 |
|  | 0045944 |
|  | 0045893 |
|  | 0061614 |
|  | 0045646 |
|  | 0006357 |
|  | 0035019 |
|  | 0000790 |
|  | 0005654 |
|  | 0005634 |
|  | 0005667 |
|  | 0003677 |
|  | 0043565 |
|  | 0002573 |
|  | 0006355 |
|  | 0001944 |
|  | 0006917 |
|  | 0000979 |
|  | 0006351 |
|  | 0001077 |
|  | 0005515 |
|  | 0006366 |
|  | 0000982 |
|  | 0001078 |
|  | 0003705 |
|  | 0001205 |
|  | 0001047 |

---

|  |  |
| --- | --- |
| KEGG ID | hsa:6688 |
|  | mmu:20375 |
|  | rno:366126 |

---

|  |  |
| --- | --- |
| Organism | Homo sapiens {Organism urn:agi-taxid:9606} |
|  | Mus musculus {Organism urn:agi-taxid:10090} |
|  | Rattus norvegicus {Organism urn:agi-taxid:10116} |
|  | Mus musculus |
|  | Homo sapiens |
|  | Rattus norvegicus |

---

|  |  |
| --- | --- |
| Mouse chromosome position | 2 50.44 cM |
|  | 2 47.5 cM |

---

|  |  |
| --- | --- |
| OMIM ID | 165170 |

---

|  |  |
| --- | --- |
| Rat chromosome position | 3q24 |

---

|  |  |
| --- | --- |
| Hugo ID | 11241 |
|  | HGNC:11241 |

---

|  |  |
| --- | --- |
| Human chromosome position | 11p11.2 |

---

|  |  |
| --- | --- |
| Swiss-Prot Accession | P17947 |
|  | P17947.2 |
|  | P17433 |
|  | Q3U5L4 |
|  | P17433.2 |
|  | Q6BDS1 |
|  | Q6BDS1.1 |
|  | Q99L57 |
|  | Q5BK69 |

---

|  |  |
| --- | --- |
| PIR ID | S60367 |
|  | A34693 |

---

|  |  |
| --- | --- |
| GenBank ID | NC\_000011 |
|  | XM\_011520307 |
|  | XP\_011518609 |
|  | XM\_017018173 |
|  | XP\_016873662 |
|  | NM\_003120 |
|  | NP\_003111 |
|  | NM\_001080547 |
|  | NP\_001074016 |
|  | AC090559 |
|  | AC090582 |
|  | CH471064 |
|  | EAW67924 |
|  | EAW67925 |
|  | EAW67926 |
|  | JC009148 |
|  | CDL93508 |
|  | KT583843 |
|  | U34046 |
|  | AAC50247 |
|  | AK310065 |
|  | BI519776 |
|  | BI909430 |
|  | BM561863 |
|  | BP271276 |
|  | CD368431 |
|  | X52056 |
|  | CAA36281 |
|  | P17947 |
|  | NC\_000068 |
|  | XM\_011239397 |
|  | XP\_011237699 |
|  | XM\_017316733 |
|  | XP\_017172222 |
|  | NM\_011355 |
|  | NP\_035485 |
|  | XM\_006499038 |
|  | XP\_006499101 |
|  | AL691450 |
|  | CH466519 |
|  | EDL27526 |
|  | AA239990 |
|  | AA789819 |
|  | AK142246 |
|  | BAE24993 |
|  | AK153524 |
|  | BAE32064 |
|  | AK154945 |
|  | BAE32942 |
|  | AK155085 |
|  | BAE33036 |
|  | BC003815 |
|  | AAH03815 |
|  | CJ187167 |
|  | L03215 |
|  | AAB59699 |
|  | M32370 |
|  | AAA40024 |
|  | M38252 |
|  | AAA40110 |
|  | X17463 |
|  | CAA35502 |
|  | P17433 |
|  | NC\_005102 |
|  | XM\_006234532 |
|  | XP\_006234594 |
|  | NM\_001005892 |
|  | NP\_001005892 |
|  | AC\_000071 |
|  | AABR07052865 |
|  | AAHX01023465 |
|  | AAHX01023466 |
|  | AAHX01023467 |
|  | CH473949 |
|  | EDL79507 |
|  | AB154364 |
|  | BAD35169 |
|  | BC091185 |
|  | AAH91185 |
|  | Q6BDS1 |
|  | XP\_011518610 |
|  | CAM17566 |
|  | AAI11380 |
|  | HG503770 |
|  | AABR06024710 |
|  | ABBA01060720 |
|  | AMYH02024729 |
|  | AMYH02024728 |
|  | HG503768 |
|  | HG503769 |
|  | AAHY01018365 |
|  | ABBA01060716 |
|  | AAHY01018367 |
|  | AAHY01018366 |
|  | ABBA01060719 |
|  | ABBA01060717 |
|  | ABBA01060718 |
|  | XM\_011520305 |
|  | XM\_011520308 |
|  | XM\_011520306 |
|  | BC111379 |
|  | AC\_000024 |
|  | AC\_000143 |
|  | NC\_018922 |
|  | XP\_011518608 |
|  | XP\_011518607 |

---

|  |  |
| --- | --- |
| Swiss-Prot ID | SPI1\_HUMAN |
|  | SPI1\_MOUSE |
|  | SPI1\_RAT |

---

|  |  |
| --- | --- |
| Cell Localization | Nucleus |

---

|  |  |
| --- | --- |
| Ensembl ID | ENSG00000066336 |
|  | ENSP00000227163.4 |
|  | ENST00000227163.8 |
|  | ENSP00000367799.3 |
|  | ENST00000378538.7 |
|  | ENSMUSG00000002111 |
|  | ENSMUSP00000002180.7 |
|  | ENSMUST00000002180.7 |
|  | ENSRNOG00000012172 |
|  | ENSRNOP00000016306.4 |
|  | ENSRNOT00000016306.6 |
|  | ENSP00000227163 |
|  | ENSP00000367799 |
|  | ENSRNOP00000016306 |
|  | ENST00000378538 |
|  | ENSRNOT00000016306 |
|  | ENST00000227163 |
|  | ENSMUSP00000002180 |
|  | ENSMUST00000002180 |

---

|  |  |
| --- | --- |
| MGI ID | MGI:98282 |
|  | 98282 |

---

|  |  |
| --- | --- |
| RGD ID | 1359607 |

---

|  |  |
| --- | --- |
| Unigene ID | Hs.502511 |
|  | Rn.230674 |
|  | Mm.1302 |

---

|  |  |
| --- | --- |
| Homologene ID | 2346 |

---

|  |  |
| --- | --- |
| IPI ID | IPI00018333 |
|  | IPI00827738 |
|  | IPI00116335 |
|  | IPI00464883 |

---
